# Quantitative ultrasound does not identify patients with an inflammatory disease at risk of vertebral deformities

**DOI:** 10.1186/1471-2474-9-72

**Published:** 2008-05-20

**Authors:** A Caroline Heijckmann, Bianca Dumitrescu, Arie C Nieuwenhuijzen Kruseman, Piet Geusens, Bruce HR Wolffenbuttel, Jolanda De Vries, Marjolein Drent, Maya SP Huijberts

**Affiliations:** 1 Department Internal Medicine, Division of Endocrinology, University Hospital Maastricht, The Netherlands; 2 Department of Internal Medicine, Hospital Bernhoven Veghel/Oss, The Netherlands; 3 Department of Rheumatology, University of medicine and Pharmacy ''Çarol Davila", Bucharest, Romania; 4 Department of Rheumatology, University Hospital Maastricht, The Netherlands; 5 Biomedical Research Institute, University Hasselt, Belgium and Nutrition and Toxicology Research Institute Maastricht (NUTRIM), The Netherlands; 6 Department of Endocrinology, University Medical Center Groningen and University of Groningen, The Netherlands; 7 Department of Medical Psychology, Tilburg University and St. Elisabeth Hospital Tilburg, The Netherlands; 8 Department of Respiratory Medicine, University Hospital Maastricht, The Netherlands; 9 Sarcoidosis Management Team, University Hospital Maastricht, The Netherlands

## Abstract

**Background:**

Previous studies from our group have shown that a high prevalence of vertebral deformities suggestive of fracture can be found in patients with an inflammatory disease, despite a near normal bone mineral density (BMD). As quantitative ultrasound (QUS) of the heel can be used for refined assessment of bone strength, we evaluated whether QUS can be used to identify subjects with an inflammatory disease with an increased chance of having a vertebral fracture.

**Methods:**

246 patients (mean age: 44 ± 12.4 years) with an inflammatory disease (sarcoidosis or inflammatory bowel disease (IBD)) were studied. QUS of the heel and BMD of the hip (by dual X-ray absorptiometry (DXA)) were measured. Furthermore lateral single energy densitometry of the spine for assessment of vertebral deformities was done. Logistic regression analysis was performed to assess the strength of association between the prevalence of a vertebral deformity and BMD and QUS parameters, adjusted for gender and age.

**Results:**

Vertebral deformities (ratio of <0.80) were found in 72 vertebrae of 54 subjects (22%). In contrast to the QUS parameters BUA (broadband ultrasound attenuation) and SOS (speed of sound), T-score of QUS and T-scores of the femoral neck and trochanter (DXA) were lower in the group of patients with vertebral deformities. Logistic regression analysis showed that the vertebral deformity risk increases by about 60 to 90% per 1 SD reduction of BMD (T-score) determined with DXA but not with QUS.

**Conclusion:**

Our findings imply that QUS measurements of the calcaneus in patients with an inflammatory condition, such as sarcoidosis and IBD, are likely of limited value to identify patients with a vertebral fracture.

## Background

Osteoporosis is a skeletal disease characterized by low bone mass and microarchitectural deterioration resulting in increased bone fragility and hence susceptibility to fracture [1,2]. The benchmark for the diagnosis of osteoporosis is the assessment of bone mineral density (BMD) with dual energy X-ray absorption (DXA) [2], as it is well established that the risk of future fracture rises with the decline of BMD. However, low BMD alone is not the only determinant of fracture risk [3] and it is evident that assessment of fracture risk should encompass all aspects of risk and not be guided exclusively by results of bone mineral density measurements [4]. In addition, in several conditions BMD evaluation provides a modest prediction of fracture risk. For example, the use of glucocorticoids (GCs) is a substantial risk factor for future fractures, which is largely independent of BMD [5,6].

We reported recently that a high prevalence of vertebral deformities suggestive of fracture can be found in patients who are considered at risk for secondary osteoporosis due to an inflammatory disease, such as sarcoidosis and inflammatory bowel disease (IBD), despite a near normal BMD [7]. This may imply that bone strength is decreased in patients with inflammatory diseases, and that changes in bone microarchitecture rather than low BMD result in an increased fracture risk [8,9].

The last years there has been increasing interest in Quantitative Ultrasound (QUS) methods for refined assessment of bone strength [10]. This noninvasive technique may assess microarchitecture and elasticity in addition to bone mineral density [11]. Several studies have demonstrated that QUS of the heel can predict fracture comparable to and independent of spine and femur BMD, and that it can be used to identify patients with higher risk [12-15]. Compared with DXA, QUS is less expensive, portable, does not require specially trained personnel and does not employ ionizing radiation.

To evaluate whether QUS can indeed be used to identify subjects likely to have a vertebral fracture irrespective of changes in BMD, we performed QUS on our series of subjects with sarcoidosis and IBD [7] and compared the results with results of vertebral fracture assessment and BMD measurements with DXA.

## Subjects and methods

### Patients

Between January 2002 and July 2003, all patients with inflammatory bowel disease or sarcoidosis who had a disease duration of at least one year, and attended the outpatient clinic of the University Hospital Maastricht, were asked to participate in this cross-sectional study. Sixteen patients with known causes of bone mass abnormalities, such as renal failure, thyroid dysfunction, alcoholism, long-term anticoagulant use and ankylosing spondylitis were excluded. Thirty-six patients were excluded because of the use of bisphosphonates or hormone replacement therapy.

Finally, 246 patients were included (mean age: 44 ± 12.4 years) of which 87 were diagnosed with sarcoidosis and 159 with inflammatory bowel disease. All patients were Caucasians and diagnosed with sarcoidosis according to the WASOG guidelines [16], based on consistent clinical features and results of an analysis of bronchoalveolar lavage fluid [17] or with CD (n = 95) or UC (n = 64) on clinical grounds using endoscopic and/or radiological evidence, and by histological investigation of mucosal biopsies and/or surgical specimens when available. For confirmation of the CD diagnosis the Lennard-Jones criteria [18] and for UC the Truelove and Witts criteria [19] were applied.

The clinical records of all patients were reviewed. Demographic, clinical and treatment data of these patients are summarized in table 1. No patients were on bisphosphonates.

**Table 1:** Demographic, treatment variables and clinical risk factors in the study patients (n = 246).

**Variable**	**Total group (n = 246)**
Age (years)	44 ± 12.4
Males/premenopausal women/postmenopausal women	109/103/34 (44/42/14)
Body mass index (kg/m^2^)	25.5 ± 4.7
Sarcoidosis/CD/UC	87/95/64 (35/39/26)
Disease duration (years)	6 (1–36)
GC use never/previous/current	74/124/48 (30/50/20)
Daily dose GC current group	12.9 (2.5–39)
Fracture > 50 years, number	2/83 (2)
Vertebral deformity by DXA	54 (22)
Low body weight (< 60 kg)	44 (18)
Low physical activity index ≤ 5	53 (22)
Mother with hip deformity	16 (7)

Data are given as mean ± SD, median (range) or number (%). Abbreviations: CD, Crohn's disease; UC, ulcerative colitis; GC, glucocorticoid; DXA, dual-energy X-ray absorptiometry.

Patients were evaluated according to a standard protocol that included questionnaires related to known clinical risk factors for osteoporosis (weight below 60 kg, hip fracture in the mother, history of fractures after age 50, menopausal status and severe immobilization) [20], calcium intake, physical activity [21], measurement of height and weight and measurement of BMD. Glucocorticoid therapy was evaluated by means of a patient questionnaire and verified using all the records of the patient's pharmacist. Informed consent was obtained from all participants and this study was approved by the ethical committee of the hospital.

### Bone mineral density and morphometry

QUS and DXA measurements were performed. QUS was performed in the left calcaneus using a Sahara device (Hologic, Waltham, MA, USA). This equipment measures the broadband ultrasound attenuation (BUA) (dB/MHz) and the speed of sound (SOS) (m/sec) in a fixed region of interest in the central calcaneal zone. The device combines the values of BUA and SOS to yield a parameter known as the "quantitative ultrasound index" (QUI) or stiffness, based on the following equation: QUI = 0.41 * (BUA + SOS) -571. The QUI is also expressed as a T-score (reference data were those provided by the manufacturer). The heel of each patient was measured three times with complete repositioning between measurements. The definitive result was the mean of these three measurements. The coefficient of variation (CV) of the QUI was 1.4%. The instrument was subjected to daily quality control using a phantom provided by the manufacturer.

BMD of the hip was measured by dual energy X-ray absorption (DXA, Hologic QDR 4500, NHANES-III reference group). The hip was measured in the standard projection, and results are reported for femoral neck and trochanter. Standard procedures supplied by the manufacturer for scanning and analysis were performed. Calibration with the manufacturer's spine phantom and quality control analysis was performed daily. The CV for BMD measurements was 1.0 %. Furthermore, after bone density measurement a lateral single energy densitometry of the thoracic and lumbar spine for vertebral fracture assessment (VFA) was performed (also called Morphometric X-ray absorptiometry (MXA)) [22]. The scans obtained were analyzed twice by one trained operator (BD) (intra-observer coefficient of variation: 0.85), using the quantitative method of Genant [23]. The observer was blinded to the T-score values and to the values of the first set of measurements. After visual examination six points were placed on each vertebral body from T4 to L4. From these points three vertebral heights were measured anterior (Ha), mid (Hm) and posterior (Hp); On the basis of the average score of these morphometric measurements ratios were calculated and a prevalent vertebral deformity was defined as a reduction of height of 20 % or more (Ha/Hp; Hm/Hp and Hp/Hp below) [23]. Severity of deformities was assessed according to the method of Genant [23]. A score of '0' was assigned to normal, non-fractured vertebra; '1' for a mild deformity (20–25% reduction in anterior, middle or posterior vertebral height); '2' for a moderate deformity (25–40% reduction) and '3' for a severe deformity (>40% reduction).

### Statistical analysis

Student t-tests, chi-square tests, and one-way ANOVAs were used, depending on the variables and subgroups tested. Logistic regression analyses was performed to assess the strength of association between the prevalence of a vertebral deformity (dependent variable) and BMD and QUS parameters (BMD femoral neck *or *BMD trochanter *or *QUI), adjusted for gender and age (covariates). A p-value < 0.05 was considered statistically significant. Analyses were performed with SPSS version 12.0.

## Results

As summarized in table 1, our series consisted of 103 pre-menopausal women, 34 post-menopausal women, and 109 men. The mean age (± SD) of this group of patients was 44 ± 12.4 years and this was similar in both sarcoidosis and IBD. With QUS, the broadband ultrasound attenuation (BUA) value was higher in men than in women (78 ± 16 versus 73 ± 15 dB/MHz, p < 0.005). No differences between the sexes of the other QUS parameters were found. The T- or Z-scores of FN or trochanter determined with DXA were not different between the sexes as well.

**Figure 1:**
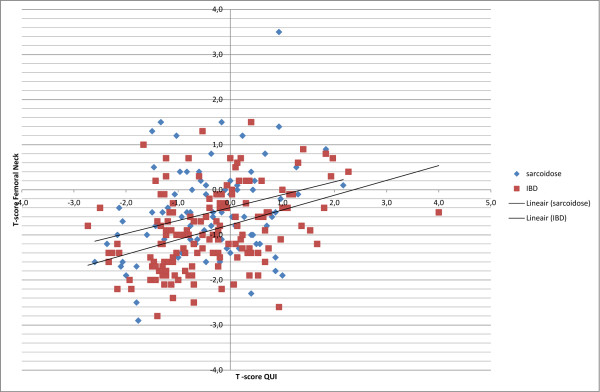
**T-score Femoral neck (DXA) versus T-score QUI (QUS)**. DXA, dual energy X-ray absorption; QUI, quantitative ultrasound index; QUS, quantitative ultrasound; IBD, inflammatory bowel disease.

T-scores of femoral neck and/or trochanter determined with DXA were in the osteopenic (T-score < -1 and > -2.5) or osteoporotic (≤ -2.5) range in respectively 50% and 2% of the patients studied, in total in 52%. In contrast, QUS of the calcaneus revealed a T-score below -1 in 32% of the patients. Correlations between DXA and QUS T-scores were r = 0.35 for the T-score of the quantitative ultrasound index (QUI) with the T-score of the femoral neck (p < 0.001) (figure 1) and r = 0.36 for the T-score QUI with the T-score of the trochanter (p < 0.001).

Clinical non-vertebral fractures had occurred in two postmenopausal women. Vertebral deformities with VFA (ratio of < 0.80) were found in 72 vertebrae of 54 subjects (22%) with a higher prevalence in men (32%) than in women (14%). Sixty-one of these were wedge and 9 biconcave deformities. Two crush deformities were seen. Multiple vertebral deformities were observed in 6% of the entire cohort and 7% had one or more moderate or severe deformities.

In table 2 data of BMD measurements with DXA and QUS in the group of patients with or without vertebral deformities are summarized. Relative to the patients without vertebral deformities, those with vertebral deformities were on average older. The former group comprised more women, the latter more men. T-scores but not Z-scores of the femoral neck and trochanter (DXA) were lower in the group of patients with vertebral deformities. The T-score of the calcaneus (QUS) was also lower in this group of patients. No differences for the other ultrasound parameters were found between the groups with or without vertebral deformities. Furthermore, no differences were found in clinical risk factors, for the different diseases, GC use, disease duration, BMI, physical activity, calcium intake, current use of calcium and/or vitamin D supplements, aminosalicylates, immunosuppressive medication, and budenoside.

**Table 2:** Bone variables in patients with and without any vertebral deformity, measured morphometrically.

	Without deformity (192)	With any deformity (54)	**All**	P*
DXA variables				
Femoral neck (g/cm^2^)	0.80 ± 0.12	0.76 ± 0.10	0.79 ± 0.11	0.006
• T-score	-0.66 ± 0.9	-1.16 ± 0.8	-0.77 ± 0.9	<0.001
• Z-score	-0.13 ± 1.0	-0.43 ± 0.9	-0.20 ± 1.0	0.06
Trochanter (g/cm^2^)	0.72 ± 0.13	0.69 ± 0.10	0.71 ± 0.13	0.2
• T-score	-0.21 ± 1.0	-0.51 ± 0.8	-0.27 ± 1.0	0.04
• Z-score	0.05 ± 1.1	-0.16 ± 0.8	0.00 ± 1.0	0.2
				
QUS variables				
BUA (dB/MHz)	76 ± 16	73 ± 14	75 ± 16	0.2
SOS (m/s)	1545 ± 89	1531 ± 99	1542 ± 92	0.3
QUI	103 ± 45	94 ± 16	101 ± 41	0.1
T-score	-0.34 ± 1.1	-0.65 ± 0.9	-0.41 ± 1.0	0.04

Data are given as mean ± SD or number (%); * p between patients with and without vertebral deformity;

Abbreviations: DXA, dual energy X-ray absorption; QUS, quantitative ultrasound; BUA, broadband ultrasound attenuation; SOS, speed of sound; QUI, quantitative ultrasound index.

Table 3 gives odds ratios (OR) per unit T-score reduction (per SD) for any vertebral deformity for the three separate regression analyses. The vertebral deformity risk increases by about 60 to 90% per 1 SD reduction determined with DXA but not with QUS. When both BMD of the femoral neck and QUI are entered simultaneously in the regression analysis the respective OR's are 1.81 (1.18 – 2.75, p = 0.006) for BMD-FN and 1.09 (0.77 – 1.56, p = 0.623) for QUI.

**Table 3:** Odds ratios* for any vertebral deformity of various bone measurements adjusted for gender and age in patients with an inflammatory disease

	**OR**	***95 % CI***	***P***
BMD femoral neck	1.88	1.26–2.81	0.002
BMD trochanter	1.63	1.12–2.37	0.01
QUI	1.31	0.95–1.81	Ns

Abbreviations: OR, odds ratio; CI, confidence interval; BMD, bone mineral density; QUI, quantitative ultrasound index.

* OR per one unit T-score reduction (per SD)

## Discussion

Our study shows that in a group of patients with an inflammatory disease as sarcoidosis and inflammatory bowel disease (IBD) none of the QUS variables had added value to recognize patients with a prevalent vertebral deformity suggestive of fracture.

In several other studies QUS has been compared with DXA in patients with IBD. Robinson and coworkers [24] studied 100 patients with Crohn's disease (CD) and 52 age-matched controls and found lower values of both BUA and SOS in CD. The correlation between BUA and BMD-values determined at the hip and spine with DXA was, however, insufficient to recommend QUS as a screening tool. In another study, 53 patients with CD and 57 with ulcerative colitis (UC) were included and QUS variables (BUA and SOS) were compared with DXA-measurements of the hip and lumbar spine [25]. Although this study also revealed a correlation between the QUS variables and DXA (r = 0.50 to 0.67), the agreement between measurements in individual patients was poor. Similar observations were made in two other studies on patients either with CD or UC [26,27]. No studies with QUS have been performed in patients with sarcoidosis.

A shortcoming of all the reported studies on the value of QUS in IBD is of course that they used BMD determined by DXA as gold standard, assuming that fracture risk increases with a decrease in BMD as in subjects without inflammatory conditions. No assessment of clinical and prevalent vertebral fractures was done. In our series, vertebral deformities suggestive of non-clinical fractures were found in 22% of patients. The T-scores of these patients determined by both DXA and QUS were on average lower than those in patients without vertebral deformities. Although there was a correlation between T-scores determined with QUS and DXA, this correlation was moderate, as found by others [28]. In addition, the calculated Odds ratios for any vertebral deformity per unit T-score reduction was increased for BMD of the hip (DXA) but not for the QUI of the calcaneus. This supports the view that the predictive value of QUS in patients with inflammatory conditions is poor. On the other hand, a limitation of our series is the moderate number of patients with one or more deformities suggestive for vertebral fracture. It is therefore possible that our study did not have adequate power to delineate an association between QUS and vertebral fractures.

Our findings are in contrast with several large prospective studies that have shown that QUS of the calcaneus can predict fracture risk nearly as good as DXA [12-14]. These studies are, however, all studies in elderly women and involve prediction of clinical (mainly non-vertebral) fractures. Kanis explored the relationship between QUS-determinations at the phalanges with age and the probability of symptomatic vertebral fractures and concluded that the 10-year probability of clinical vertebral fractures above the age of 45 increased for each SD decrease in measurement of SOS and fast wave amplitude (RR 1.7, respectively 2.4/SD) [29]. Studies on morphometric vertebral deformities and QUS parameters are, however, scarce. In 764 postmenopausal women (mean age 73 ± 6.4 years) the prevalence of nontraumatic vertebral fractures assessed with DXA was compared with an age matched control group with normal morphometry and this study showed that heel QUS enabled discrimination of women with fracture from those without [30]. The same findings were reported in another study in postmenopausal women with rheumatoid arthritis [31]. On the other hand, other studies revealed no differences in patients with and without a prevalent vertebral deformity. In one cross-sectional study in 551 post-menopausal women (mean age 65.2 ± 13.1) receiving chronic glucocorticoid therapy a high prevalence of asymptomatic morphometric vertebral fractures was found (37%), without any difference in QUS measurements between patients with and without deformities [32]. This indicates that if QUS may be of any value to predict fracture risk, this will be in postmenopausal women and not in the type of patients included in our study.

Although the T-scores of patients with vertebral deformities of our series were lower than in those without, the Z-cores were not different. This means that the differences in T-score are likely due to differences in age rather than differences in disease activity. In addition, despite the fact that the T-scores were lower, they were certainly not diagnostic for osteoporosis, indicating that in inflammatory conditions, an increased fracture risk is due to changes in bone strength that are not combined with changes in BMD [7].

## Conclusion

In our hands QUS measurements of the calcaneus in patients with an inflammatory condition, such as sarcoidosis and IBD, were not associated with prevalent vertebral deformities and are therefore likely not of value to recognise patients at risk for fracture. Hence, we feel that both BMD measurement with DXA and vertebral fracture assessment are better methods to identify such patients. Follow-up studies are, however, needed to substantiate this view.

## Competing interests

Performance of the study was supported by an unrestricted educational grant from Merck Sharpe and Dome, the Alliance for Better Bone Health and Eli Lilly. The authors declare that they have no competing interest.

## Authors' contributions

ACH coordinated study concept and design, performed acquisition, analysis and interpretation of data and prepared the manuscript. BD analyzed the scans for vertebral fracture assessment and took part in manuscript preparation. ACNK and PG took part in interpretation of data and preparation of the manuscript. BHRW was involved in study concept and design and preparation of the final manuscript. JDV performed analysis, interpretation of data and preparation of the final manuscript. MD did acquisition of subjects, interpretation of data and preparation of the final manuscript. MSPH was involved in analysis and interpretation of data and preparation of the manuscript. All authors read and approved the final manuscript.

## Pre-publication history

The pre-publication history for this paper can be accessed here:


